# A Case Report of Thalamic Infarction after Lumbar Drain: A Unique Cause of Perioperative Stroke?

**DOI:** 10.1155/2019/8764706

**Published:** 2019-06-09

**Authors:** Daniel N. Kianpour, Thomas M. Nguyen, Arthur M. Lam

**Affiliations:** ^1^Swedish Neuroscience Institute, Seattle, WA, USA; ^2^University of California San Diego, USA

## Abstract

In the case presented, a patient has an unexplained episode of hypertension during aneurysm clipping. Following the procedure, the patient was discovered to have bilateral thalamic infarctions unrelated to the vascular location of the aneurysm. After a review of the case, it becomes apparent that intracranial hypotension caused by lumbar over drainage of cerebrospinal fluid (CSF) is the likely cause of both the episode of intraoperative hypertension and the thalamic infarcts. It is often presumed that having an open dura protects against intracranial hypotension and subsequent herniation. We present this case to suggest that opening the dura might not be protective in all cases and anesthesiologists must pay particular attention to the rate of CSF drainage. Lumbar CSF drainage is a technique frequently employed during neurological surgery and it is important for anesthesiologists to understand the signs, symptoms, and potential consequences of intracranial hypotension from rapid drainage.

## 1. Introduction

Lumbar cerebrospinal fluid drainage (CSF) drainage is a commonly used approach during cerebrovascular and skull base neurosurgical procedures to aid in brain relaxation and surgical exposure. With a closed dura, one well-known complication of lumbar drainage is the abrupt increase in an aneurysm's transmural pressure gradient caused by acute CSF drainage which can lead to catastrophic rebleeding [1]. A much less recognized complication is “brain sagging”; acute drainage of CSF can cause a rapid decrease in intracranial pressure resulting in some degree of transtentorial herniation [2]. Once the dura has been opened, rapid changes in intracranial pressure by drainage are diminished and thought to limit the brain sagging seen with a closed dura. Intraoperatively, brain sagging and herniation can manifest with Cushing's response of hypertension and bradycardia which can be underrecognized given the number of confounding variables in this setting. The case presented demonstrates an occurrence of presumed herniation syndrome from CSF drainage during elective unruptured anterior communicating artery aneurysm clipping causing significant intraoperative hypertension and subsequent bilateral thalamic infarctions. This case has been prepared following the CARE guidelines and methodology for medical case reports [3].

## 2. Case Presentation

A 64-year-old, 87 kg female presented for clipping of a 4 mm wide neck unruptured saccular anterior communicating artery aneurysm which was discovered incidentally during the evaluation of headaches and memory difficulties. Her past medical history was significant for remote breast carcinoma, hypertension, obstructive sleep apnea, and gastroesophageal reflux disease. She was a lifelong nonsmoker and notable preoperative medications included aspirin, furosemide, and propanolol. Preoperative imaging had no evidence of prior infarcts. After induction of anesthesia, a right internal jugular central venous catheter and radial arterial catheter were placed and maintenance of anesthesia was performed with a combination of 0.5 MAC sevoflurane, propofol 50 mcg/kg/min, and remifentanil 0.125 mcg/kg/min infusions. Mannitol 1 gram per kilogram for a total of 80 grams was administered. The neurosurgeon placed an 80 cm closed-tip, barium impregnated lumbar drainage catheter with a 0.7 mm inner diameter (Medtronic USA, REF 46419) at the L3-4 level prior to the start of surgery.

The surgery commenced and was progressing unremarkably. Prior to opening of the dura, 20 mL of CSF was drained over 15 minutes per neurosurgeon request. After dural opening and during dissection of the aneurysm, the surgeon requested the lumbar drain to be opened allowing further drainage of CSF. After approximately 15 minutes, the blood pressure sharply increased over the course of 1 to 2 minutes from a baseline systolic blood pressure of 130 mmHg to over 205 mmHg with an associated decrease in heart rate from 60 bpm to 50 which resolved over the course of minutes. This acute hypertension was treated with a number of interventions including 250 mg propofol and 1 mcg/kg remifentanil boluses aimed at treating light anesthesia as well as a bolus of 7.5 mg labetalol. At the time of the hypertensive event, the propofol, remifentanil, and Sevoflurane dosing had been stable and unchanged for over an hour. There was also no change in the level of surgical stimulation at this time as the dura had been incised and the neurosurgeons were using the operative microscope to expose the aneurysm. After the aneurysm was successfully clipped, the lumbar drain was closed and the surgery was completed without further episodes of hypertension. A total of 60 mL of CSF was drained via the lumbar drain during the case in addition to the losses from the surgical field.

**Figure 1:**
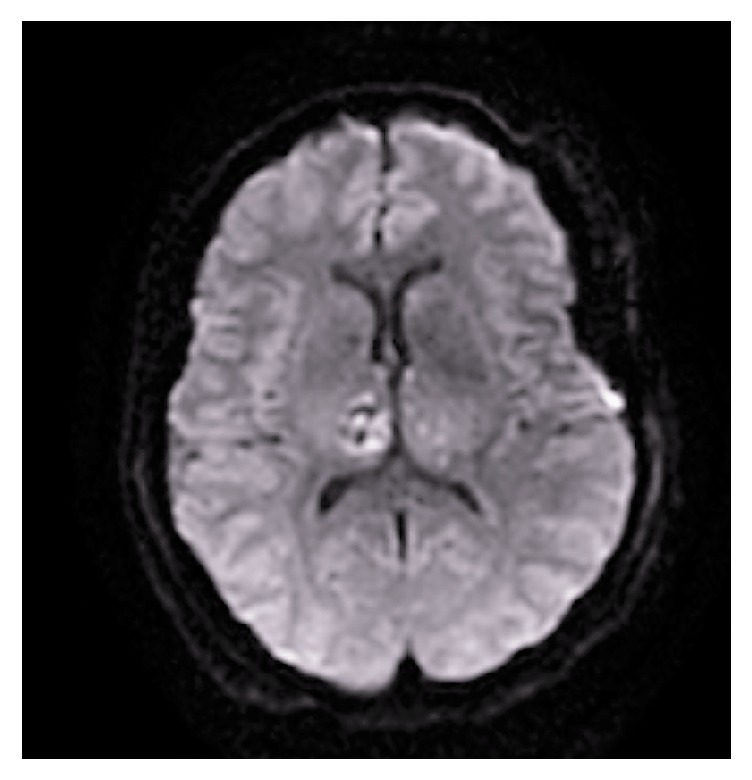
Diffusion weighted image MRI from postoperative day 1.* Note*. Increased signal intensity in right basal ganglia with punctate increases on the left indicating acute infarcts.

At conclusion of the operative procedure, the patient remained comatose and unresponsive despite an hour in the operating room awaiting emergence from anesthesia. A postoperative head CT was obtained prior to transport to the intensive care unit which demonstrated mild cerebral edema and borderline inferior transtentorial herniation, but no significant hemorrhage or focal abnormalities. She was then transported to the Neurocritical Care Unit intubated and ventilated. Initial arterial blood gas analysis on arrival to the ICU did not reveal a cause to her delayed emergence (pH 7.35, pCO2 47 mmHg, pO2 323 mmHg, glucose 204 mg/dL, and sodium 137 mEq/L). She did not receive any benzodiazepines during the case, but did receive 50 mcg of fentanyl on induction and 1 gram of levetiracetam in addition to the propofol, remifentanil, and Sevoflurane maintenance. Initial neurological exam demonstrated midline and equal 4 mm pupils which were reactive to light bilaterally. She withdrew to painful stimuli in all four extremities. On postoperative day (POD) 1, her mental status continued to be depressed with a Glasgow Coma Scale of 7T (E2:V1T:M4). That day, an MRI was obtained which revealed bilateral thalamic infarctions on the diffusion weighted imaging which can be seen in Figure 1. Supportive care was continued and over the course of several days, her mental status slowly improved to GCS of 10 (E4:V1:M5) on POD 5. This allowed for safe extubation that day. She continued to improve and was alert and oriented to person, place, time, and situation with some memory and attention difficulties by POD 16. A timeline of her in-hospital recovery can be found in Table 1. She was discharged home with referrals for home physical, occupational, and speech therapy on POD 19. She continued her gradual neurological improvement and by over a year postoperatively, Neurology records indicated she was living independently and her Montreal Cognitive Assessment score had recovered to a normal 27/30.

## 3. Discussion

The case presented describes a patient who had sudden and severe unexplained hypertension during aneurysm clipping without obvious changes in anesthetic depth or surgical stimulation, but did have an open and draining lumbar CSF catheter. Although the causality between CSF drainage and hypertension is not clear, there are two important features which argue for it as the etiology of the observations made. Firstly, this case demonstrates the phenomenon of systemic hypertension related to CSF drainage. Although not commonly seen with the relatively low flow rates of modern lumbar drainage catheters, it was well recognized with previous types of CSF drainage devices. In his 1975 letter to the editor of* Anaesthesia*, John Barker notes that with the use of an 18-gauge malleable needle, “Drainage of cerebrospinal fluid is controlled by a gate clamp in order to avoid the intense arterial pressor response and cardiac arrhythmia which can occur with free flow” [4]. With rapid drainage of CSF, the brain begins to sag, thus causing transtentorial herniation and subsequent reflexive systemic hypertension [5]. Secondly, our patient had findings of bilateral thalamic infarctions on postoperative MRI, demonstrated in Figure 1. Transtentorial herniation is known to cause compression of the posterior cerebral arteries and potentially subsequent arterial and venous infarctions in its territory which includes the thalamus [6, 7]. In one case series of 32 patients with lumbar drainage catheters, Roland describes a case of infarction caused by “kinking” of the PCA at the tentorium cerebelli [8]. In another series of patients with persistent unconsciousness and evidence of intracranial hypotension after uneventful neurological surgery, 16 of 16 patients displayed radiologic evidence of injury to bilateral thalamic or basal ganglia structures [9]. The combination of severe hypertension and the subsequent finding of thalamic infarction is strong evidence that intracranial hypotension from CSF drainage contributed to the findings observed.

This case illustrates an important point: dural opening may not protect against intracranial hypotension and subsequent Cushing's response as well as commonly thought. CSF over drainage should be included in the differential diagnosis of any unexplained hypertension in the presence of an open lumbar drainage catheter after other causes of hypertension such as aneurysm rupture and light anesthesia are excluded. It is also imperative to alert our neurosurgical colleagues if we notice a possible Cushing's response whether being from intracranial hypotension or other causes such as brain compression.

Although this episode of hypertension cannot be definitively linked to brain sagging and transient transtentorial herniation, the evidence strongly suggests that it is the likely etiology and anesthesiologists should be aware of this phenomenon. With the utilization of endovascular treatment for cerebral aneurysms, future anesthesiologists will continue to have decreased exposure to open surgery and the nuances associated with it. It will be increasingly important to better describe in the literature these nuances where previous generations such as that of John Barker received from clinical practice.

## Figures and Tables

**Table 1:** Timeline of in-hospital neurologic recovery.

*Postoperative Day (POD)*	*0*	*1*	*2*	*3*	*5*	*10*	*15*	*19*
*Glasgow Coma Scale (Total)*	7	8	9	10	11	14	14	15
*Best Eye Response*	2	3	3	4	4	4	4	4
*Best Verbal Response*	1T	1T	1T	1T	1	4	4	5
*Best Motor Response*	4	4	5	5	6	6	6	6
*Wakefulness/Orientation *	Unarousable	Arousable	Arousable	Arousable	Alert Disoriented	Alert, Oriented times 2^a^	Alert, Oriented times 1^b^	Alert, Oriented times 4^c^
*Communication*	Unable to Assess	Unable to Assess	Unable to Assess	Unable to Assess	Expressive Aphasia	Expressive Aphasia	Nonsensical Speech	Loss of Fluency

^a^Oriented to person and situation. ^b^Oriented to person only. ^c^Oriented to person, place, time, and situation.
